# A major QTL corresponding to the *Rk* locus for resistance to root-knot nematodes in cowpea (*Vigna unguiculata* L. Walp.)

**DOI:** 10.1007/s00122-015-2611-0

**Published:** 2015-10-08

**Authors:** Bao-Lam Huynh, William C. Matthews, Jeffrey D. Ehlers, Mitchell R. Lucas, Jansen R. P. Santos, Arsenio Ndeve, Timothy J. Close, Philip A. Roberts

**Affiliations:** Department of Nematology, University of California, Riverside, CA 92521 USA; Bill and Melinda Gates Foundation, Seattle, WA 98102 USA; Department of Botany and Plant Sciences, University of California, Riverside, CA 92521 USA

## Abstract

**Key message:**

**Genome resolution of a major QTL associated with the*Rk*locus in cowpea for resistance to root-knot nematodes has significance for plant breeding programs and R gene characterization.**

**Abstract:**

Cowpea (*Vigna unguiculata* L. Walp.) is a susceptible host of root-knot nematodes (*Meloidogyne* spp.) (RKN), major plant-parasitic pests in global agriculture. To date, breeding for host resistance in cowpea has relied on phenotypic selection which requires time-consuming and expensive controlled infection assays. To facilitate marker-based selection, we aimed to identify and map quantitative trait loci (QTL) conferring the resistance trait. One recombinant inbred line (RIL) and two F2:3 populations, each derived from a cross between a susceptible and a resistant parent, were genotyped with genome-wide single nucleotide polymorphism (SNP) markers. The populations were screened in the field for root-galling symptoms and/or under growth-chamber conditions for nematode reproduction levels using *M. incognita* and *M. javanica* biotypes. One major QTL was mapped consistently on linkage group VuLG11 of each population. By genotyping additional cowpea lines and near-isogenic lines derived from conventional backcrossing, we confirmed that the detected QTL co-localized with the genome region associated with the *Rk* locus for RKN resistance that has been used in conventional breeding for many decades. This chromosomal location defined with flanking markers will be a valuable target in marker-assisted breeding and for positional cloning of genes controlling RKN resistance.

**Electronic supplementary material:**

The online version of this article (doi:10.1007/s00122-015-2611-0) contains supplementary material, which is available to authorized users.

## Introduction

Root-knot nematodes (*Meloidogyne* spp.) (RKN) are among the most devastating soil-borne crop pests worldwide, causing annual losses amounting to billions of dollars in global agriculture (Abad et al. 2008). They parasitize plant root systems and thus directly affect the uptake of water and nutrients needed for normal plant growth and reproduction. Their infection of plant roots can also be components of disease complexes with other pathogens including vascular diseases such as Fusarium wilt and root rots (Roberts et al. 1995). Cowpea (*Vigna unguiculata* L. Walp.) is a common host of RKN; it is grown in the USA and on larger scales in semi-arid regions of Africa and other warm to hot regions where RKN is prevalent (Ehlers and Hall 1997; Olowe 2004; Sawadogo et al. 2009). Several RKN species are known to attack cowpea, among which *M. incognita* and *M. javanica* are most widespread (Fery and Dukes 1980; Roberts et al. 2005). Due to cost and safety limitations on the use of chemical nematicides, nematode management in cowpea includes alternative strategies involving crop rotations and host resistance (Roberts et al. 2005). Using nematode-resistant cowpea cultivars as a grain crop or as cover crops in cropping systems can promote yield and suppress nematode populations in the soil during crop rotation (Roberts et al. 2005).

Quantitative inheritance studies have indicated that RKN resistance in cowpea involves different additive genes (Ehlers et al. 2000b; Fery and Dukes 1980; Fery et al. 1994; Roberts et al. 1996). Of these, the *Rk* gene, designated by Fery and Dukes in 1980, is effective against different RKN isolates and thus has been deployed in modern cultivars via conventional breeding using phenotypic selection (Ehlers et al. 2002, 2009). Until now, there has not been a marker-based definition of the *Rk* locus to facilitate indirect selection of the resistance trait in breeding programs. Breeders have relied on field, greenhouse and controlled environment techniques for resistance phenotyping; however, these approaches are expensive, time-consuming and often hindered by inaccuracies due to variations in phenotyping assays. Field screening for resistance to nematodes involves the use of isolated plots that are managed to maintain high and uniform nematode population densities through systematic crop rotation protocols with susceptible crops in non-screening years. Plants grown in infested field plots are visually rated for extent of root-galling. Controlled inoculation screens using greenhouse-grown potted plants or in seedling growth pouches grown in controlled environment chambers have also been developed and are more accurate than field screens but are also more labor-intensive (Ehlers et al. 2000b; Omwega et al. 1988; Roberts et al. 1996). While some phenotyping is necessary in marker-assisted selection breeding, development of markers for indirect selection of nematode resistance has many advantages, especially when high-throughput genotyping platforms are available, as they are now for cowpea (Huynh et al. 2015; Muchero et al. 2009). In this study we report on the identification of a major QTL in the cowpea genome which contains the *Rk*-gene trait determinant, with defined flanking markers based on genic-SNPs. This paves the way for deployment of a more efficient breeding strategy using genetic markers.

## Materials and methods

### Mapping populations

Three cowpea segregating populations used in genetic mapping included (1) 87 recombinant inbred lines (RILs) from the cross CB27 × 24-125B-1, (2) 170 F2:3 families from the cross IT84S-2049 × UCR779 and (3) 132 F2:3 families from the cross IT93K-503-1 × UCR779. CB27 is a California Blackeye dry grain variety bred by University of California-Riverside (UCR) (Ehlers et al. 2000a) while IT84S-2049 and IT93K-503-1 are breeding lines developed by the International Institute of Tropical Agriculture (IITA, Nigeria), all of which are highly resistant to RKN. The breeding line 24-125B-1 was developed by the Institute de Recherche Agricole pour le Development (IRAD, Cameroon) and UCR 779 is a Botswana cowpea landrace donated by de Moy (Colorado State University) in 1987, both of which are highly susceptible to *M. incognita* and *M. javanica*.

### Nematode isolates

Three root-knot nematode isolates were used for resistance screening. The *M. incognita* isolates ‘Project 77’ and ‘Beltran’ originally isolated from cotton and lima bean fields, respectively, in the San Joaquin Valley, California, were characterised previously to be avirulent on cowpeas with resistance conferred by the *Rk* gene (Roberts et al. 1995). The *M. javanica* isolate ‘811’ is aggressive on cowpea plants with *Rk* resistance, rendering the resistance response partially effective, and was isolated from a cowpea field in southern California. All isolates have been maintained on greenhouse-grown susceptible tomato plants (Thomason and McKinney 1960).

### Resistance phenotyping: egg-mass production

The RIL population CB27 × 24-125B-1 and parents were assayed for RKN resistance using a seedling growth-pouch inoculation system described in Ehlers et al. (2000b) and visualized in Atamian et al. (2012). In brief, cowpea seeds from each RIL were germinated in a petri dish. Each germinated seed was then transferred into one pouch. The pouches were watered daily with distilled water and kept in a controlled environment growth chamber with constant temperature (27 °C) and 16 h of light per day. When adequate root systems were developed (about 14 days), each pouch was inoculated with approximately 1500 second-stage juveniles of *M. incognita* isolate project 77. The nematode juvenile inoculum was prepared by hatching nematode eggs extracted from tomato roots. After inoculation, the plants were maintained in Hoagland’s growth solution (Hoagland and Arnon 1950) for 30 days and then treated with egg-mass-selective erioglaucine dye (Sigma Chemical Co., St. Louis, MO, USA) overnight. Stained egg masses on plant roots were counted with the aid of a 10×-illuminated magnifier. The experiment was arranged in a randomized complete block design with three replications, each containing one seedling per pouch per RIL. Mean number of egg masses per RIL was used to classify resistance levels.

### Resistance phenotyping: root-galling symptoms

**Fig. 1:**
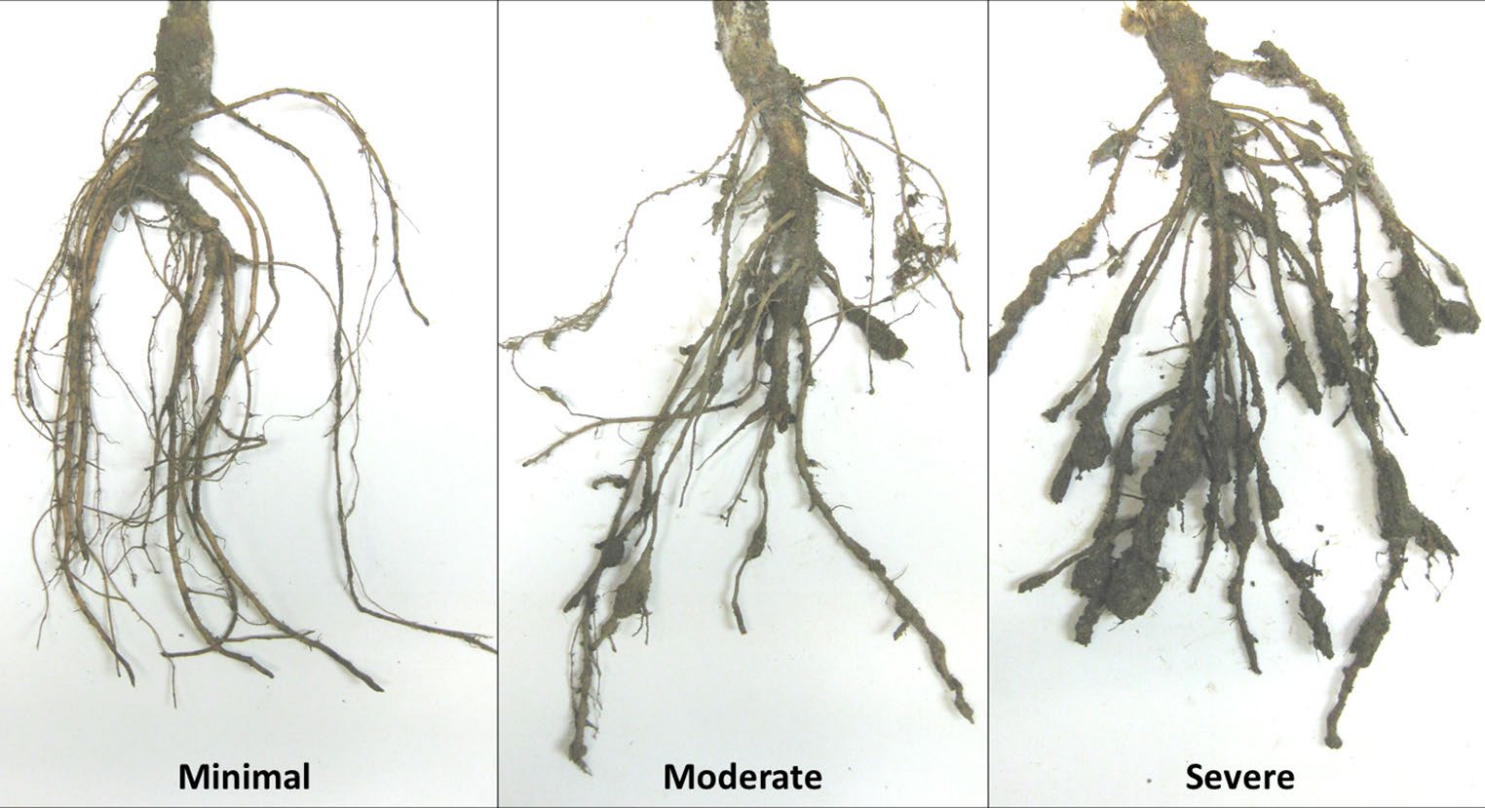
Examples of root-galling symptoms induced by root-knot nematodes observed in the phenotype screens of mapping populations grown in the field tests

The mapping populations plus parents were evaluated at the University of California South Coast Research and Extension Center (SCREC). In 2008, the CB27 × 24-125B-1 RIL population was tested with *M. javanica* isolate 811. The IT84S-2049 × UCR779 and IT93K-503-1 × UCR779 F2:3 populations were tested with *M. incognita* isolate Beltran in 2010 and 2012, respectively. In 2012, the CB27 × 24-125B-1 RILs were screened in separate field blocks, one infested with *M. incognita* isolate Beltran and the other infested with *M. javanica* isolate 811. The field sites contained root-knot nematode infestations which were established by injecting an inoculum of nematode eggs extracted from greenhouse-grown tomato plants into the root-zone of young susceptible tomato plants, followed by several years of growing susceptible tomatoes to provide high and uniform infestation levels. Nematode-induced galling symptoms on the tomato root systems were scored throughout the experimental block as a bio-assay before the cowpea experiment in each site, ensuring that the infestation levels were at least a score 7–8 using the root-gall rating chart of Bridge and Page (1980). Nematode species identification was verified by isozyme profiles and the N. Carolina host differential test of the original inocula and rechecked by these methods and with species-specific DNA primers by PCR prior to the cowpea experiments. Each RIL or F2:3 family was grown in one 1.5-m-long plot, with 20–25 plants per line or family. Uniformity and intensity of RKN infection in all experiments were confirmed on the basis of similar mean plot scores of susceptible parents (24-125B-1 and UCR779) that were grown next to the resistant parent in 5 replications (20–25 plants per replicate) throughout the experiment as positive controls for nematode infection. After 60–70 days, the plant tops were cut and each root system dug and scored for galling symptoms using a scale from 0 (no symptoms) to 9 (severe galling), modified from the rating chart of Bridge and Page (1980) (Fig. 1).

For the CB27 × 24-125B-1 RIL population, Analysis of Variance (ANOVA) was performed with the software GenStat version 11 (Payne et al. 2008). Factors in the ANOVA model were lines and block, with each field and year considered as a block. Broad-sense heritability was estimated based on the variance component attributable to variation among lines (VG) and residual variation (VE) [*h*^2^ = VG/(VG + VE)]. Simple linear correlation analysis was used to examine the phenotypic relationship between egg-mass numbers on pouch-grown root systems and root-galling symptoms measured in different field screenings. For F2:3 populations, mean score of galling symptoms per family was used to classify resistance levels.

### Linkage analyses and QTL mapping

Genotypic data of 1536 genome-wide SNP markers (Muchero et al. 2009) for the CB27 × 24-125B-1 RIL population were obtained from Lucas et al. (2011). For two F2 populations (IT84S-2049 × UCR779 and IT93K-503-1 × UCR779), F2 individuals of each population were genotyped with the Kompetitive allele-specific polymerase chain reaction (KASP) assay (LGC Genomics Ltd., Hoddesdon, UK) (Semagn et al. 2014) using 168 and 155 SNP markers, respectively. These markers were polymorphic between the two parents of each corresponding population and spaced at least 2 cM apart on the cowpea consensus genetic map (Lucas et al. 2011) [also accessible at HarvEST:Cowpea 1.33 (http://harvest.ucr.edu/)]. Linkage-map construction was performed with the software QTL IciMapping 4.0 (http://www.isbreeding.net) using the Kosambi function, RECORD ordering algorithm (Van Os et al. 2005) and alignment with the cowpea consensus genetic map.

The Inclusive Composite Interval Mapping (ICIM) method (Li et al. 2007; Wang 2009) was also performed with the software QTL IciMapping 4.0. ICIM involved three consecutive steps: (1) significant markers associated with phenotypes were selected through single marker analysis; (2) the selected markers, except for two markers flanking the current mapping interval, were used to adjust phenotypic values; and (3) the adjusted phenotypic values were used in composite interval mapping (Yang et al. 2007) for QTL identification.

### Genotyping of near-isogenic lines (NILs)

Three NILs that differ in the presence or absence of the *Rk*-resistance were developed using conventional backcrossing. In brief, the recurrent parent CB46 (homozygous resistant, *RkRk*) (Helms et al. 1991) was crossed with a highly susceptible cowpea landrace ‘Chinese Red’ (homozygous susceptible, *rkrk*), and then the F1 was backcrossed to the recurrent parent CB46 to generate BC1F1. Blind crosses were then made between BC1F1 plants with CB46, and selfed seeds (BC1F2) of each BC1F1 plant were screened with *M. incognita* isolate Project 77 in growth pouches for variation in RKN resistance based on egg-mass production, in comparison with the parents CB46 and Chinese Red. Subsequently, crosses made from heterozygous resistant BCF1 plants (*Rkrk*), whose BC1F2 segregated for resistance, were selected for the next backcross cycle. Backcrossing was repeated for six cycles followed by single seed descent and phenotyping at the F6 to select for homozygous susceptible lines. Two highly susceptible lines (Null 1 and Null 2) were genotyped with 1536 SNPs using the Illumina GoldenGate assay. Marker profiles of these lines and the original parent plants were aligned with the cowpea consensus genetic maps (Lucas et al. 2011) for identification of regions that coincided with QTLs detected in mapping populations.

## Results

### Resistance variation

**Fig. 2:**
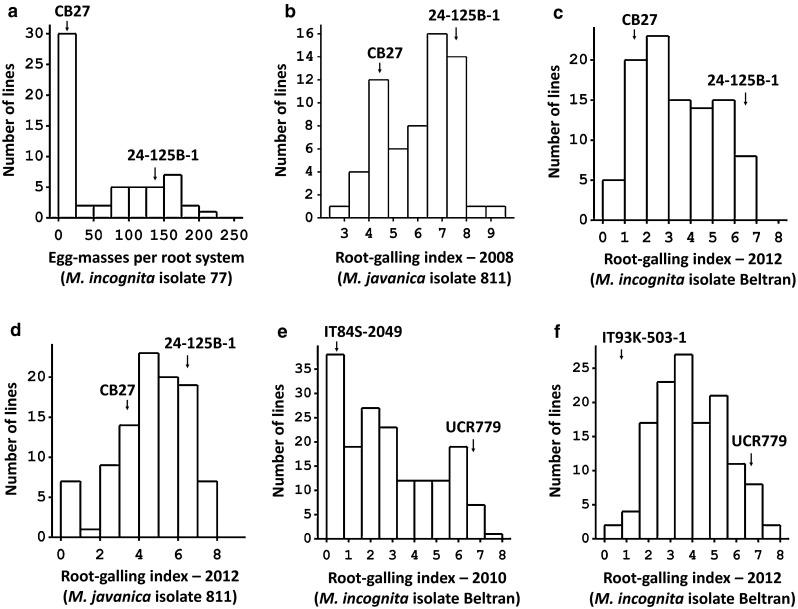
Variation in response to root-knot nematode infection within the cowpea populations (**a**–**d**) CB27 × 24-125B-1 RIL, (**e**) IT84S-2049 × UCR779 F2:3, and (**f**) IT93K-503-1 × UCR779 F2:3 phenotyped in multiple tests with different RKN isolates (in *parentheses*). Mean phenotypic values for the parents in each test are indicated by *labelled arrows*. The number of RKN egg-masses per root system was measured in inoculated growth pouches. Root-galling was indexed in infested field plots

The production of egg-masses (EM) varied widely among the CB27 × 24-125B-1 RILs grown in pouches tested with *M. incognita*, ranging from 0 to 208 mean EM per root system (Fig. 2a). The segregation appeared to follow a bi-modal distribution, with the resistant parent CB27 showing a lower mean EM number than the susceptible parent 24-125B-1. In field experiments, root-galling (RG) scores also varied widely, ranging from 0 to 9 (Fig. 2b, c, d). The distributions were skewed toward the lower phenotypic value in the *M. incognita* tests, whereas in *M. javanica* tests the distributions were skewed toward the higher phenotypic value. The RG data for the RIL population were quite repeatable across years and nematode fields, with broad-sense heritability estimated at 0.8. There were also significant correlations (*r* > 0.45, *P* < 0.001) between EM and RG data, with resistant CB27 showing lower phenotypic values than susceptible 24-125B-1 in all experiments.

Large variation in RG symptoms was also observed among F2:3 families of the crosses IT84S-2049 × UCR779 (Fig. 2e) and IT93K-503-1 × UCR779 (Fig. 2f) when grown in the field tested with *M. incognita*. Resistant parents IT84S-2049 and IT93K-503-1 showed lower RG scores than the susceptible parent UCR779. The distribution appeared skewed toward lower phenotypic values in both mapping populations.

### QTL identification

**Fig. 3:**
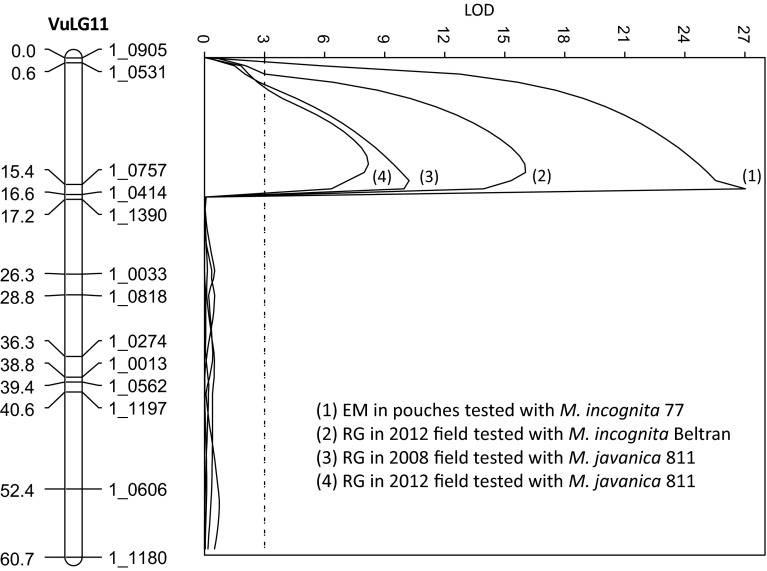
Chromosomal locations on cowpea linkage group VuLG11 of the CB27 × 24-125B-1 RIL population associated with the variation in root-knot nematode egg–mass production (EM) and root-galling symptoms (RG) in four experiments

A QTL with major effect on EM production and RG symptoms was consistently mapped in the same region on linkage group VuLG11 of the CB27 × 24-125B-1 RIL population using data from four separate RKN phenotyping assays (Fig. 3). The QTL effect was highest in the EM assay with *M. incognita*, explaining 73 % of total phenotypic variance (PVE), followed by field screens with *M. incognita* in 2012 (PVE = 71 %) and *M. javanica* in 2008 (PVE = 59 %) and 2012 (PVE = 52 %) (Table 1). The favourable (low EM numbers and RG scores) allele was contributed from the resistant parent CB27.

**Table 1:** Chromosome locations associated with resistance to different root-knot nematode species and isolates in cowpea RIL and F2:3 populations phenotyped in inoculated seedling growth pouches and infested field plots

Mapping population (female × male)	Resistance trait (environments, RKN isolates)	Linkage group	Position (cM)	Flanking markers	LOD	PVE (%)^a^	Add^b^	Dom^c^	D/A^d^
RIL (CB27 × 24-125B-1)	Egg masses (growth pouches, *M. incognita* 77)	VuLG11	16	1_0757–1_0414	27.0	72.9	−57.6	NA	NA
	Gall score (field 2012, *M. incognita* Beltran)	VuLG11	14	1_0531–1_0757	16.0	70.9	−1.5	NA	NA
	Gall score (field 2008, *M. javanica* 811)	VuLG11	15	1_0531–1_0757	10.2	59.2	−1.0	NA	NA
	Gall score (field 2012, *M. javanica* 811)	VuLG11	13	1_0531–1_0757	8.2	52.4	−1.4	NA	NA
F2:3 (IT84S-2049 × UCR779)	Gall score (field 2010, *M. incognita* Beltran)	VuLG11	19	1_0905–1_0414	60.8	83.1	−2.7	−0.7	0.26
F2:3 (IT93 K-503-1 × UCR779)	Gall score (field 2010, *M. incognita* Beltran)	VuLG11	14	1_0886–1_0757	29.3	64.5	−1.8	−0.4	0.22

^a^Percentage of variance explained

^b^Additive effects: negative values indicate that alleles from the susceptible male parents contributed to higher phenotypic values

^c^Dominance effects: negative values indicate that the mean of lines heterozygous for the QTL is lower than the phenotypic value half way between the mean of lines homozygous for one allele and that of lines homozygous for the alternative allele (*NA* not applicable in the RIL population)

^d^The dominance/additive ratios showing gene action modes as partial dominance (D/A = 0.21–0.80)

**Fig. 4:**
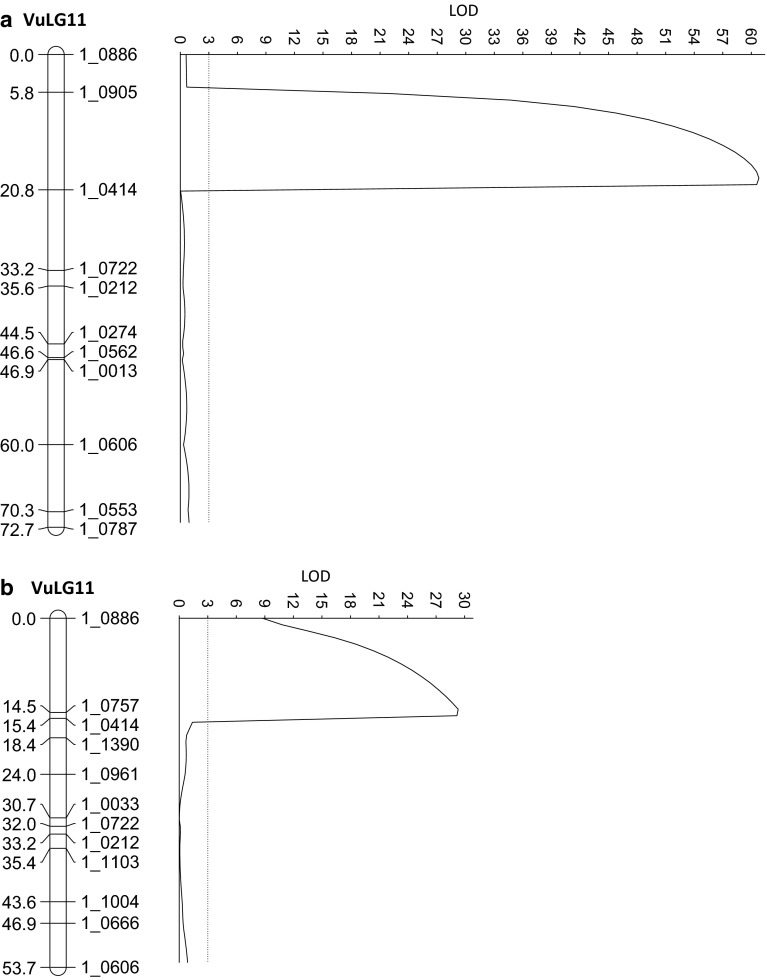
Chromosomal locations on linkage group VuLG11 of the **(a)** IT84S-2049 × UCR779 and **(b)** IT93K-503-1 × UCR779 F2 populations associated with the variation in root-galling symptoms expressed in the F2:3 families grown in *field plots* infested with *M. incognita* isolate Beltran in 2010 and 2012, respectively

A major QTL associated with RG symptoms was also mapped on linkage group VuLG11 of each F2:3 population when assayed with *M. incognita* in the field in 2010 (Fig. 4). The QTL effect was higher in the IT84S-2049 × UCR779 population (PVE = 83 %) than in the IT93K-503-1 × UCR779 population (PVE = 65 %) (Table 1; Fig. 4); in both F2:3 populations, the QTL showed partial dominance, and lower RG scores were conferred by the presence of the IT84S-2049 and IT93K-503-1 alleles. SNP markers nearest to this QTL (1_0757 and 1_0414) also flanked the major QTL detected in the CB27 × 24-125B-1 RIL population (Fig. 3).

### The *Rk* location

**Fig. 5:**
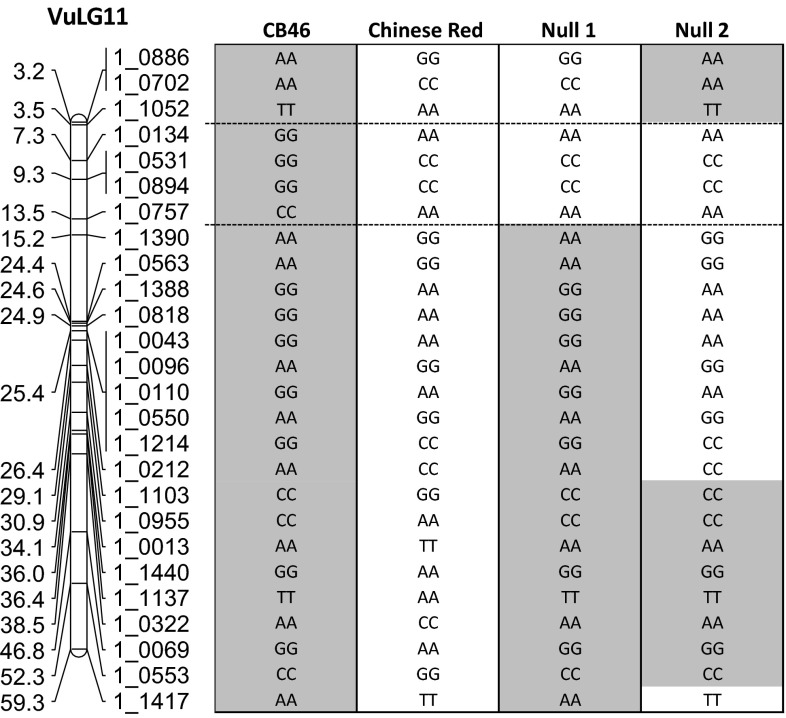
Chromosomal location on linkage group VuLG11 of the cowpea consensus genetic map (Lucas et al. 2011) associated with *Rk*-type resistance as revealed by SNP genotyping of two susceptible NILs (*Null 1* and *Null 2*) and their recurrent parent CB46 (*Rk* resistant) and susceptible donor Chinese Red. SNP markers that are polymorphic between CB46 (*grey*) and Chinese Red (*clear*) are indicated. The full dataset of 1536 genome-wide SNP markers is provided in the Supplementary File. The genomes of the two susceptible NILs were homozygous for the CB46 background except for a common region (between the *two dashed lines*) that was homozygous for the susceptible donor alleles. Markers in this region (1_0134, 1_0531 and 1_0757) also flank the major QTL mapped on VuLG11 of the RIL and F2 populations (Figs. 3 and 4)

Genotyping of the resistant CB46 and two susceptible NILs (Null 1 and Null 2) revealed that the NILs were almost completely homozygous for the CB46 background except for a common region of about 6 cM on VuLG11 of the cowpea consensus genetic map that was homozygous for the susceptible donor (Chinese Red) alleles (Fig. 5 and Supplementary File). This region contains SNP markers (1_0531, 1_0894 and 1_0757) that also flank the major QTL mapped on VuLG11 in the three mapping populations (Figs. 3 and 4).

## Discussion

The skewed or bi-modal phenotypic distributions observed for resistance traits, based on nematode reproduction and root-galling symptoms (Fig. 2), support the presence of a major QTL which was detected in each mapping population. Given their common proximity to marker 1_0414, the three QTLs mapped in separate populations (Figs. 3 and 4) could be confined to a single multi-allelic locus, hereafter designated *QRk*-*vu11.1*. By genotyping of *Rk* and non-*Rk* NILs derived from conventional backcrossing, we confirmed that *QRk*-*vu11.1* corresponds to the *Rk* locus, which was first designated by Fery and Dukes (1980) and has been used in conventional breeding of resistant cowpea for a long time (Ehlers et al. 2000a, 2009; Hall et al. 2003; Hall and Frate 1996). Based on cowpea consensus genetic maps (Lucas et al. 2011), *QRk*-*vu11.1* is not in the same locations of previously published QTLs for resistance to other biotic traits such as fungal diseases (Muchero et al. 2011; Pottorff et al. 2012, 2014), viruses (Ouédraogo et al. 2002), the parasitic weed Striga (Ouédraogo et al. 2012) and insects (Huynh et al. 2015; Lucas et al. 2012). *QRk*-*vu11.1* could be deployed in breeding programs by SNP-genotyping to select directly for the *Rk* locus, minimizing the need for laborious controlled phenotype assays.

The two susceptible null-*Rk* lines were developed following the conventional scheme applied in RKN resistance breeding (Ehlers et al. 2002). It took more than 5 years from the original cross between the recurrent parent CB46 and the susceptible donor Chinese Red to develop BC6F6 lines for field evaluation; each backcross cycle required at least 5 months for blind crossing, collecting selfed-seeds and then phenotyping the selfed-seeds to decide which cross to advance. Genotyping of BC6F6 lines (Fig. 5, Supplementary File), however, revealed substantial linkage drag in some lines that extended many centimorgans beyond QTL-flanking markers. Since a cM may be hundreds of kilobases in length (Dohm et al. 2012; Zhang et al. 2012), with the 620 Mb cowpea genome having an average genetic map length of 680 cM (Lucas et al. 2011; Muchero et al. 2009), conventional backcrossing potentially introduced many ‘unwanted’ genes from resistance donors. With marker-assisted backcrossing, desirable recombinants with minimal linkage drag could be selected based on both flanking and adjacent markers around *QRk*-*vu11.1*. Furthermore, combining this foreground selection approach with background selection for the recurrent parent genotype using available genome-wide markers could expedite the breeding process.

The nematode isolates used in this study represent two main RKN species (*M. incognita* and *M. javanica*) that cause substantial damage to cowpea in the USA and most cowpea-growing regions worldwide (Petrillo et al. 2006). Based on genetic mapping, the QTL *QRk*-*vu11.1* exhibited a major effect on resistance against both species, albeit with a lower effect on *M. javanica* (Table 1). This is in contrast to soybean where QTLs conferring resistance to *M. incognita* and *M. javanica* were mapped on different chromosomes (Tamulonis et al. 1997; Xu et al. 2013), indicating a broad functionality of the *Rk* locus in the cowpea system. Variation in the effectiveness of the *Rk*-based resistance to different isolates of these two species in cowpea has been documented (Petrillo et al. 2006; Petrillo and Roberts 2005). This variation reflects differences in virulence among *M. incognita* populations, with evidence of selection for virulence to *Rk* in fields routinely planted with cowpea cultivars carrying the *Rk* gene, while some *M. javanica* populations are more aggressive on *Rk* plants than avirulent *M. incognita*. Nevertheless, the broad utility of this resistance to most root-knot nematode populations in cowpea production areas is highly valuable for nematode management programs. Furthermore, our positioning of the *Rk* locus on the cowpea genetic map provides an important step in the comparative characterization of other RKN resistance genes in cowpea which hitherto have been identified by non-marker-based inheritance studies (Ehlers et al. 2000b, 2002; Roberts et al. 1996).

Previous studies showed that *Rk*-resistance did not suppress root penetration by infective juveniles of *M. incognita*, but blocked female development and reproduction (Das et al. 2008). Expression profiling revealed hundreds of genes showing differential expression between the *Rk* and non-*Rk* NILs following *M. incognita* infection (Das et al. 2010). The discovery of the QTL *QRk*-*vu11.1* location in the cowpea genome region containing gene *Rk* with defined markers will enable fine mapping of *QRk*-*vu11.1* and provide a framework for identifying the *Rk* trait determinant. Using the new knowledge reported here, we are currently testing candidate genes in the QTL region to determine the specific determinant of the *Rk*-based nematode resistance.

## Electronic supplementary material

Supplementary material 1 (XLSX 126 kb) Genome-wide SNP profiles of near-isogenic lines and parents that differ in the presence or absence of the *Rk*-resistance
